# Visuospatial training has positive effect on language abilities in children with Delirium diagnoses and inclusion of delirium-specific

**DOI:** 10.1192/j.eurpsy.2021.1342

**Published:** 2021-08-13

**Authors:** S. Kiselev

**Affiliations:** Clinical Psychology, Ural Federal University, Ekaterinburg, Russian Federation

**Keywords:** specific language impairments, visuospatial training, visuospatial abilities

## Abstract

**Introduction:**

It was shown that children with specific language impairments (SLI) have deficits not only in producing and understanding language but also in visuospatial abilities (Kiselev et al., 2016). We assume that training programs that are aimed to develop the visuospatial abilities can help children with SLI.

**Objectives:**

The goal of this study was to assess the impact of visuospatial training on the language abilities in 6–7 years old children with SLI.

**Methods:**

The participants were 20 children aged 6–7 years with SLI. Children were randomly assigned to the intervention and comparison group. Children from intervention group participated in 8 weeks of visuospatial training. This programme trains the child to do different visuospatial exercises both on motor and cognitive level. This programme is built on the conceptual framework derived from the work of Luria’s theory of restoration of neurocognitive functions (Luria, 1963, 1974). We used the subtests from Luria’s child neuropsychological assessment battery to assess language abilities in children before and after the intervention period.

**Results:**

Analysis of covariance tested the effect of visuospatial training programme on five language subtest from Luria’s child neuropsychological assessment battery. Group differences (p<.05) were found for subtest that assess understanding prepositions that describe the spatial relations between objects. Posttest mean for the intervention group were significantly (p<.05) greater than the control group.

**Conclusions:**

It can be assumed that visuospatial training in children with SLI benefits specific language abilities for understanding sentences with spatial prepositions.

